# Characterizing multi-criteria decision analysis, risk stratification, and hotspot analysis methods to optimise health policymaking and evidence translation for medicines in low- and middle-income countries: A scoping review

**DOI:** 10.12688/gatesopenres.16402.1

**Published:** 2026-10-15

**Authors:** Luke E. Norris, Tessa Rose Cornell, Patricia N. Okorie, Lily-May Hudson, Louise A. Kelly-Hope

**Affiliations:** 1 University of Liverpool Institute of Infection Veterinary and Ecological Sciences, Liverpool, UK; 2 Medical Entomologist Consultant, Kanata, Ottawa, Canada

**Keywords:** multi-criteria decision analysis, risk stratification, hotspot analysis, medicines, drugs, vaccines

## Abstract

**Background:**

Implementing new medicines or health interventions in lower-income and middle-income countries (LMICs) may be complex and challenging, and there is a need for practical structured approaches and tools to support the translation of evidence into practice, including the implementation of evidence-informed interventions and health policy making.

**Objective:**

This scoping review aimed to characterise three data analytical methodologies - multi-criteria decision analysis (MCDA), risk stratification, and hotspot analysis - to evaluate their use in guiding the implementation of medicines for human health in LMICs between 2010-2025.

**Methods:**

Scientific articles were searched in databases PubMed and Scopus following guidelines from the Preferred Items for Systematic Reviews and Meta-Analysis (PRISMA). Data were extracted in relation to the study aims, geographic scope and scale, author affiliations, disease focus, medicine type (categorised as oral drugs or vaccines), target population, medicine implementation phase, use of templates or tools, and main steps of the methodological process. In addition, grey literature was searched by reviewing conference abstracts, national public health websites and journals.

**Results:**

From the 4,920 articles screened, a total of 40 articles (MCDA=8, risk stratification= 22, hotspot analysis=10) were included from 28 countries across five global regions, mostly Africa. Most articles focused on communicable infectious diseases, and drugs (oral regimens) and vaccines in the pre-implementation stage at a sub-national level. The processes and techniques used for each analytical method varied widely, limiting comparability. However, common methodological steps were identified and used to construct preliminary templates to summarise the processes involved in applying each methodology and support their potential adaptations for decision-making around medicine implementation.

**Conclusion:**

These analytical methodologies provide a structured basis for further developing practical templates and decision-support tools to enhance the medicine translation; however, efforts are needed to raise awareness, strengthen capacity, and facilitate greater uptake.

## Introduction

Implementing new medicines or health interventions in lower-income and middle-income countries (LMICs) may be complex and challenging for many reasons, such as constraints in infrastructure, limited access to digital technology, shortage of capacity, and the lack of practical, context-appropriate guidelines and frameworks. ^1^^–^^3^ This is particularly concerning in view of the high and disproportionate burden of communicable and non-communicable diseases and/or infectious and non-infectious diseases affecting millions of vulnerable people in these low resource settings. ^4^^,^^5^ Strategic implementation of interventions is essential to improve health outcomes, alleviate health system pressures, and make progress toward the United Nations Sustainable Development Goals (SDGs).

The research and development of essential medicines, including developing new treatments and improving existing ones, are critical to the global health agenda, by combating disease and improving quality of life. ^1^ This aligns with the SDG Good Health and Well-Being (SDG 3) targets that support research and development, and universal access to affordable medicines and vaccines for diseases, particularly those affecting LMICs (Target 3.b); set out to achieve universal health coverage, including access to quality essential health-care services, and medicines and vaccines for all (Target 3.8); and end epidemics of communicable diseases such as AIDS, tuberculosis and malaria (Target 3.3). ^5^^,^^6^

Bridging the research findings of new medicines or expanding existing health interventions to different regions, population groups, or purposes is challenging, especially in LMICs. ^3^ There is a need to translate the complex evidence into guidelines or briefings to inform local policy and practices and provide key information on the intervention or processes for non-experts in the intervention or processes such as donors. Functionally, it is critical that countries have practical approaches that support national programmes, or by proxy, the World Health Organization (WHO) guidelines. A single evidence translation cycle, leading to LMIC decisions based on research findings, depends on clear guidelines and tools, including well-designed visual aids and decision-support tools to enhance understanding and support the implementation process. ^7^

Data analytical frameworks and methods provide a structured approach that may be developed into user-friendly formats to guide national programmes and communicate to donors the value of the investments in applying these methods. ^2^^,^^3^ Multi-criteria decision analysis (MCDA), risk stratification, and hotspot analysis are three examples of methods that have been used for different public health purposes, including evidence translation of research findings. They complement related processes such as Health Technology Assessments in evaluating interventions and guiding decision makers. ^8^^–^^10^

MCDA is tool and extension of decision theory that combines individual (often conflicting) criteria into one overall appraisal to support decision-making. ^11^^–^^13^ MCDA helps decision-makers choose between multiple options while considering multiple objectives. MCDA has been increasingly integrated in Health Technology Assessment processes, including through model frameworks derived from MCDA principles, such as the Evidence and Value: Impact on DEcision-Making (EVIDEM) framework. ^14^^,^^15^

Risk stratification in public health has broad scope but may be defined as a process of delineating individuals or populations into different risk levels (e.g., high, medium, low) based on a combination of multiple factors such as clinical characteristics, demographics, environment and socioeconomic status. ^16^^,^^17^ Risk stratification may be used to make decisions regarding resource allocation and interventions.

Hotspot analysis uses geographic information and spatial analysis techniques to identify statistically significant clusters of high values (hotspots) and low values (cold spots) within a dataset. ^18^^,^^19^ The most common use in epidemiology is to identify areas of high disease burden or transmission risk or medicine coverage. The identification of disease or coverage hotspots and/or cold spots can play important roles in decision-making regarding intervention planning and policymaking.

The extent to which the three data analytical methodologies have been used in LMICs to implement or monitor the impact of new or existing medicines for human health has not been explored collectively. Such an assessment may provide key insights into their local use and capacity, disease focus, data types, analytical steps, and processes. Therefore, understanding the common processes and methodological steps used across these approaches may help identify how they can be applied in different contexts. However, accessible examples describing the use and application of these methods may be limited, highlighting the need for practical frameworks to support stakeholders in understanding and planning the use of these prioritisation techniques, rather than to provide comprehensive, definitive, or validated workflows.

The aim of this scoping review was to characterise the MCDA, risk stratification, and hotspot analysis methodologies, evaluate their use, and determine whether broad templates of the processes could be developed as decision-support tools to optimise human health policymaking and evidence translation for medicines in LMICs.

## Materials and methods

### Study design

The scoping review of scientific and grey literature was conducted using the Preferred Items for Systematic Reviews and Meta-Analysis (PRISMA) guidelines for scoping reviews. ^20^ A complete PRISMA-Scoping Review (PRISMA-ScR) checklist is available in the S1 PRISMA-Checklist file in the Dryad Data Repository (DOI: 10.5061/dryad.kwh70rzjp).

Data were summarised by three main areas noted here, with a brief description of the focus, which are listed fully as research questions in the data item and extraction section below.

1. Article and report attributes – publication year, geographic scope, authors of research.
2. Methods and data reported – human disease focus, medicine type, target population, implementation phase, geographical scale, use of templates of methods.
3. Approach and finding – aims, templates and tools, main steps in process.

### Working definitions

**Data analytical methods**

The following working definitions were used for the three data analytical methods of interest. Definitions were developed following a preliminary literature review of studies applying these methods.

- **MCDA:** A tool and extension of decision theory that combines individual (often conflicting) criteria into one overall appraisal to support decision-makers.
- **Risk Stratification:** A method focusing on delineating populations into different risk levels based on factors such as clinical characteristics, demographics, environment, socioeconomic status.
- **Hotspot Analysis:** A method using geographic information and spatial analysis techniques to identify statistically significant clusters of high values (hotspots) and low values (cold spots) within a dataset.

**Interventions**

The World Health Organization (WHO) International Classification of Health Interventions (ICHI) defines a health intervention as “act performed for, with or on behalf of a person or a population whose purpose is to assess, improve, maintain, promote or modify health, functioning or health conditions.” ^21^ For the purposes of this scoping review, we limited health interventions to include only oral drugs and vaccines. We also define “new” health interventions as those that were in market (patented) but had not yet been introduced to an area or population.

**Low-income and middle-income countries and WHO regions**

Literature describing studies in LMICs was reviewed. The Organisation for Economic Co-operation and Development (OECD) 2022 list of countries with low-income or middle-income economies was applied to identify relevant countries. ^22^ The OECD categorised countries based on gross national income per capita, as published by the World Bank.

LMICs were grouped into WHO regions which are defined as follows: WHO African Region (AFR), WHO Region of the Americas (AMR), WHO South-East Asia Region (SEAR), WHO Eastern Mediterranean Region (EMR), WHO Western Pacific Region (WPR) ( https://www.who.int/about/who-we-are/regional-offices ).

**Human diseases and groups**

Human diseases or disease groups were categorised broadly as follows: i) infectious diseases i.e. caused by pathogens such as bacteria, viruses, parasites, ii) non-infectious diseases i.e. caused by factors such as genetics, environmental, physiology, iii) communicable diseases, subset of infectious diseases that can be transmitted from person-to-person, and iv) non-communicable (NCDs), subset of mostly non-infectious disease, caused by non-infectious factors such as genetics, environmental, physiology, ^23^ although there are also some infectious non-communicable diseases (e.g. tetanus). The number of articles examining a single disease, or multiple diseases or groups were summarised.

### Scientific literature

**Data sources and search strategy**

A literature search was conducted in two electronic databases, PubMed ( https://pubmed.ncbi.nlm.nih.gov/) and Scopus ( http://scopus.com/). The search was conducted on 5 June 2025. The search timeframe was restricted to studies published from 2010 until the search date. Search terms in Scopus were limited to the title, abstract or keywords of an article. No further search restrictions were applied in PubMed.

Separate searches were conducted across both databases for each of the three analytical methods. The search strings are available in full within the PRISMA form at the end of the document (S1 PRISMA-Checklist). ^20^ Search strings included three common search term groups applied across all analytical methods: i) disease terms (infecti* OR communicable OR “non-infecti*” OR “non-communicable”); ii) medicine/drug terms, (drug OR medicine OR pharmaceutical OR therapeutic OR vaccine OR vaccination OR inoculation OR “health technology assessment” OR HTA); iii) and low- and middle-income country terms.

In addition, specific terms were used for each analytical methods, including i) MCDA terms “multi-criteria decision analysis” OR “multiple criteria decision analysis” OR “multicriteria decision analysis” OR “multi-criteria decision making” OR “multicriteria decision making” OR “multiple criteria decision making” OR “multi-attribute decision analysis” OR “multiattribute decision making” OR “multicriteria analysis” OR “multi-criteria analysis” OR “multi-criteria-analysis” OR “multiple criteria analysis” OR “multiple-criteria analysis” OR “multiple-criteria-analysis” OR “multiple attribute decision making” OR “multi criteria decision aiding” OR MCDA OR EVIDEM OR MACBETH); ii) risk stratification terms (“stratify risk” OR “risk stratify” OR “risk prediction” OR “risk score” OR “risk index” OR “predictive model” OR “risk stratification” OR “risk adjustment” OR “risk profiling” OR “risk screening” OR “predictive modelling” OR “risk assessment” OR “risk standards” OR “risk classification”); and iii) hotspot terms (“spatial Analysis” OR “geospatial analysis” OR “geographic mapping” OR “spatial regression” OR “space time clustering” OR “spatiotemporal analysis” OR “spatio-temporal analysis” OR “risk cluster” OR “geographic cluster” OR “cluster analysis” OR “cluster mapping” OR “spatial autocorrelation” OR “hotspot analysis” OR “hot spot analysis”).

***Eligibility criteria***

The inclusion criteria include: i) utilises one or more of the following data analytical methods: MCDA, risk stratification, hotspot analysis, ii) focus is the translation of medicines/interventions for human disease(s), iii) conducted in LMICs, iv) published since 2010 and v) available in English. The exclusion criteria included: i) literature that does not meet all inclusion criteria and ii) review articles. Search results were exported into EndNote reference software ( https://endnote.com/).

***Study selection***

Search results were exported from EndNote reference software into Rayyan review software ( https://www.rayyan.ai). ^24^ Duplicates were removed using EndNote and Rayyan de-duplication functions. Remaining papers were checked manually for remaining duplicates during the review process.

Identified papers were subject to two levels of review on Rayyan review software:

1. Titles and abstracts of identified articles were reviewed by first and second reviewers (LEN, TRC and PNO). LEN and TRC reviewed MCDA articles and hotspot analysis articles, while LEN and PNO reviewed risk stratification articles. All reviews were performed independently. Literature was marked as ‘Yes’ (relevant according to eligibility criteria), ‘No’ (not relevant), or ‘Maybe’. Articles with conflicts (i.e. disagreements between first and second reviewers) and those marked as ‘Maybe’ were reviewed and resolved by a third reviewer (LAKH).
2. Literature which passed through the first stage of review was subject to full text review, using the same reviewer process as described for titles and abstracts.

### Data items and extraction

Specific research questions were developed and grouped into the following categories. In sections (1) and (2), every question applied to all three analytical methods. In question section (3) certain questions only applied to a specific analytical method.

1. **Paper and report attributes**
  - How many articles have been published since 2010?
  - What regions and countries were the focus of the studies?
  - What proportion of articles were published by authors affiliated with organizations in the regions and countries of the research focus?
2. **Methods and data reported**
  - What human diseases and type of diseases were studied?
  - What type of human medicines were included (i.e. drugs, vaccines)? Were they considered ‘new’ medicine?
  - What stage of the medicine translation process was studied? (e.g. before, during and/or after implementation activity)
  - Who were the target populations?
  - What geographical or administrative level was studied?
3. **Approach and findings – specific to each analytical method**
  For each methodology
  - What were the main aims of articles?
  - Were any templates or decision-tools, including visual images presented to highlight the process of each methodological approach? Could they be used as a basis for standardizing methodological approaches?
  - What were the main steps in the process of the frameworks/methodologies used and how were they defined? e.g.
    ○ MCDA (e.g., criteria selection and prioritization, weighting)
    ○ Risk stratification (e.g., risk score, thresholds)
    ○ Hotspot analysis (e.g., Getis-Ord Gi*, Kulldorff’s spatial scan statistic, Local Moran’s I)
  MCDA;
  - Were additional or related frameworks used or referred to? (e.g. EVIDEM)
    Risk stratification;
  - Was the approach based on or adapted from an existing study?
    Hotspot analysis;
  - What specific software were used to conduct hotspot analysis e.g. ArcGIS?

Articles which passed through full text review were subject to data extraction. Data items were extracted using three separate forms (one form for each analytical method) developed on Kobo Toolbox platform ( https://www.kobotoolbox.org/).

Data extraction results exported to Microsoft Excel files were cross-checked for accuracy and to ensure data extraction approaches were standardized between reviewers.

### Data analysis and synthesis

A scoping review flow diagram was populated for each analytical method. Each flow diagram specified the number of papers identified from the electronic database search, the number of duplicates removed, the number of papers removed on title/abstract review, the number of papers removed on full text review, and the final number of papers included in the scoping review.

Descriptive statistics summarised the characteristics of included papers, under the three main question categories of data items and key method-specific questions. Summary statistics were described in the text, tabulated, or presented by graphics.

Visual tools presented in scientific literature were exported and compared, to inform the development of more standardised tool templates.

### Grey literature

**Data sources, search strategy and eligibility criteria**

The scientific literature search was complemented by a desk-based review of the grey literature, using the same eligibility criteria and search terms. A selected number of sources were included as a proof of principal as a more comprehensive examination was beyond the scope of this study. Grey literature sources searched included the following:

1) American Society of Tropical Medicine & Hygiene (ASTMH; https://www.astmh.org/) conference abstract books (2015-2024). ASTMH conference abstracts were selected due to the conference’s open-accessibility, global scope, and a diverse range of infectious disease research. Related publications of identified abstracts were sought.
2) Targeted websites of national and regional public health institutions and academic institutions of three of the most populous countries in AFR and AMR, including national public health journals (issues published 2023-25).
3) Targeted WHO Regional Office websites ( https://www.who.int/about/who-we-are/regional-offices ).
4) The WHO Institutional Repository for Information Sharing (IRIS) ( https://www.who.int/library).
5) CABI digital library ( https://www.cabidigitallibrary.org/).

**Data items, extraction and analysis**

Data extraction and analysis for grey literature followed the general approach described above for the scientific literature. Key variables (where available) included data source, publication date, title, analytical method, geographic scope, target population, disease(s), medicine or intervention, and stage of translation. Data were manually extracted, entered into Microsoft Excel (Microsoft Corporation, Redmond, WA, USA), and summarised descriptively.

### Ethics and dissemination

The scoping review used only information from publicly available sources; therefore, institutional review board approval and consent were not required.

## Results

### Paper and report attributes

**Summary**

A total of 4,920 articles were identified across all three analytical methods, which included the 14 citations sourced separately. Following duplicate removal, 4,231 articles were included for title and abstract screening: MCDA (n = 32), risk stratification (n = 2,755), and hotspot analysis (n = 1,442). Full texts of 122 articles were screened: MCDA (n = 12), risk stratification (n = 87), and hotspot analysis (n = 21). A total of 40 full text articles were eligible for inclusion in the review: MCDA (n = 8, 5 articles sourced separately), ^25^^–^^32^ Risk Stratification (n = 22, 1 article sourced separately), ^33^^–^^54^ and Hotspot Analysis (n = 10, 1 article sourced separately). ^55^^–^^64^ See Figure 1.

**Figure 1.**
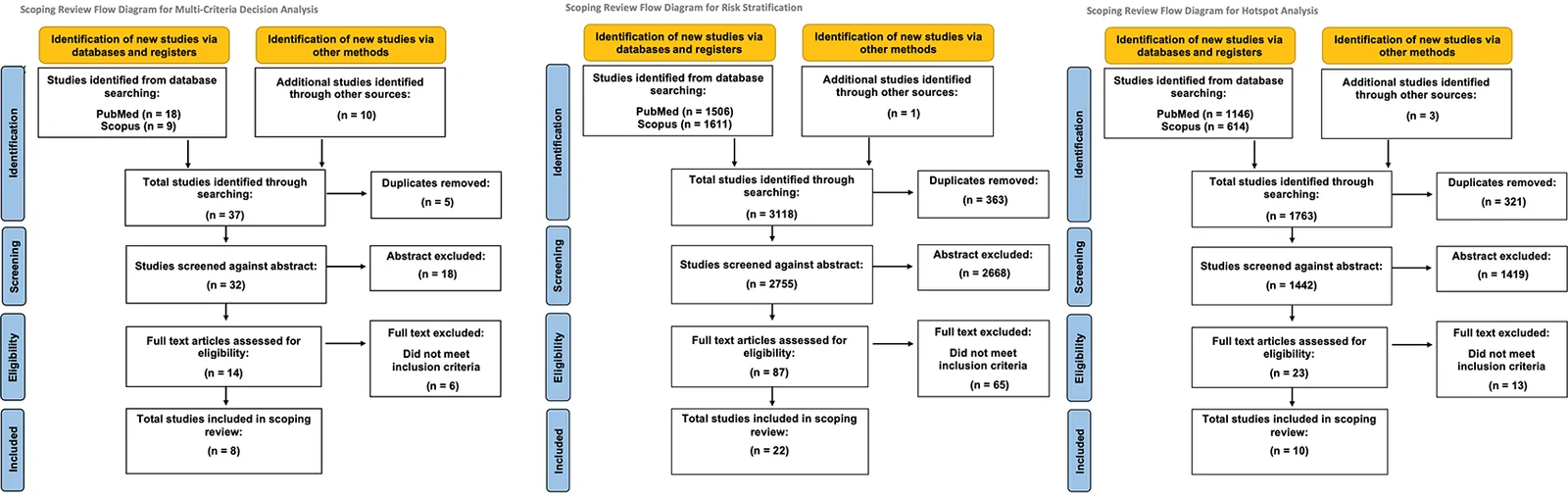
Study selection flow chart of each analytical method.

**Publication time, place, authors**

The 40 articles included were published from 2011 to 2025 with nearly two-thirds (65.0%) published since 2020 ( Figure 2A). The highest annual number of studies (n = 7) were published in 2022. MDCA articles were published between 2011-2024, risk stratification articles between 2011-2025, and hotspot analysis articles between 2012-2024. Data are summarised in the S1 Appendix in the Dryad Data Repository (DOI: 10.5061/dryad.kwh70rzjp).

**Figure 2.**
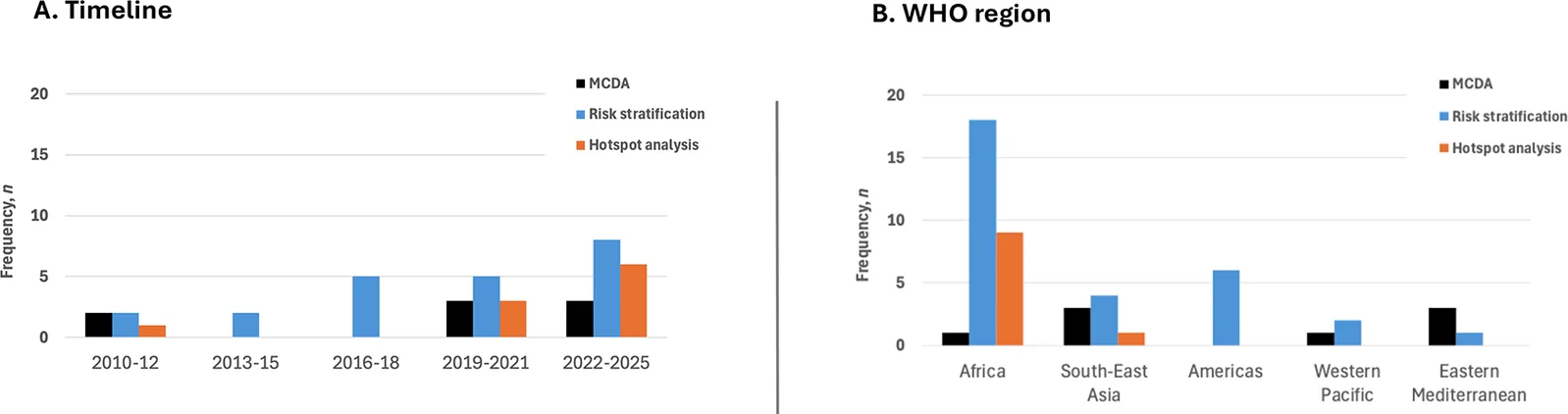
Summary graphs of data analytical articles highlighting A) Timeline B) WHO region.

The distribution of articles across WHO regions was variable ( Figure 2B). MCDA studies were conducted in four regions (AFR n = 1, SEAR n = 3, EMR n = 3, and WPR n = 1). All MCDA articles focused on their single respective regions. Risk stratification articles were conducted across five WHO regions (AFR n = 18, AMR n = 5, SEAR n = 3, and WPR n = 1). In total, 18 risk stratification articles focused on a single region, while 3 were multi-regional, and one covered 110 countries. Countries with the highest number of articles included South Africa (n = 7), Uganda (n = 6), Kenya, Malawi and Zimbabwe (n = 5 each). Hotspot analysis articles were conducted mainly in AFR (n = 9) and one in SEAR. All hotspot analysis articles focused on a single region. Ethiopia (n = 7) reported the highest number of articles. Data are summarised in the S1 Appendix in the Dryad Data Repository (DOI: 10.5061/dryad.kwh70rzjp).

Overall, 37.5% of articles reported alignment between the country affiliation of both the first or last authors and the study country (MCDA n = 4, risk stratification n = 4, and hotspot analysis n = 7). In 20.0% of articles, either the first or last author’s country affiliation was aligned with the study country (MCDA n = 2, risk stratification n = 5, and hotspot analysis = 1). In contrast, 42.5% of articles reported that the country affiliation of neither the first nor the last author were aligned with the study country (MCDA n = 2, risk stratification = 13, and hotspot analysis = 2). Data are summarised in the S1 Appendix in the Dryad Data Repository (DOI: 10.5061/dryad.kwh70rzjp).

### Analytical methods and data reported

**Human disease**

Table 1 summarises the number of articles examining a single disease or multiple diseases or groups and the categories. Four MCDA articles included multiple diseases, whereas all risk stratification and majority of hotspot analysis (n = 9) articles focused on a single disease. MCDA articles focused on infectious communicable and infectious non-communicable diseases, and one article did not specify an exact disease focus, referring collectively to ‘orphan diseases’. ^30^ All risk stratification (n = 22) and most of the hotspot analysis (n = 9) articles focused on infectious diseases, including a mixture of communicable and non-communicable diseases ( Figure 3A).

**Table 1.** Summary of disease focus and group by analytical methods.

Disease summary	MCDA	Risk stratification	Hotspot analysis *
Single-disease focus	3	22	9
Multiple-disease focus	4	0	1
Disease(s) not specified	1	0	0
Infectious - communicable	3	20	5
Infectious – non-communicable	2	2	4
Both communicable and non-communicable diseases	2	0	0

* One Hotspot Analysis article focused on multiple vaccines presentation diseases and not clear of category type.

**Figure 3.**
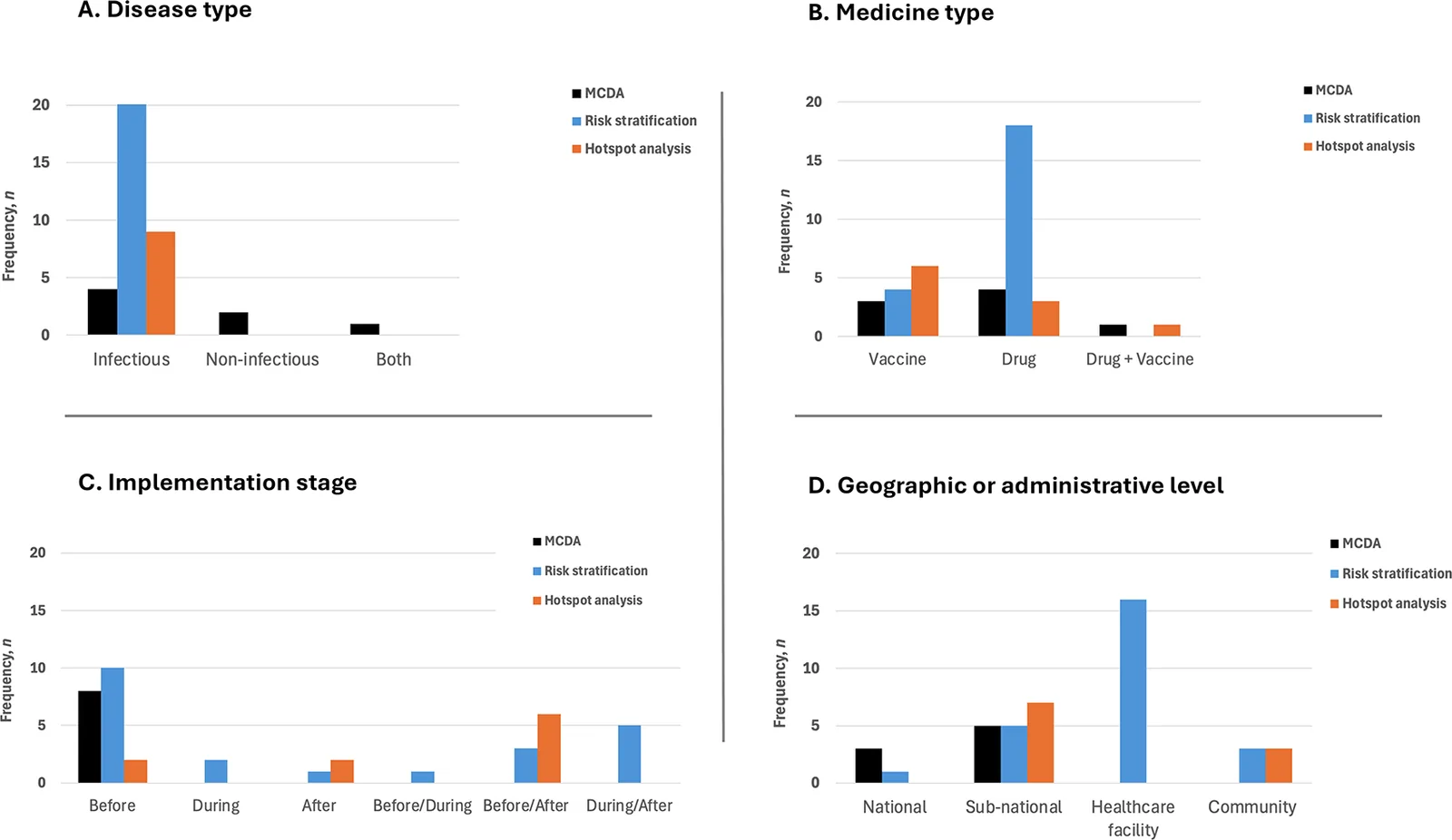
Summary graphs of data analytical articles highlighting A) Disease type B) Medicine type C) Implementation stage D) Geographical or administrative level.

Overall, 30 diseases or disease groups were reported in the articles (see S1 Appendix in the Dryad Data Repository DOI: 10.5061/dryad.kwh70rzjp for full list). The MCDA articles included a total of 21 human diseases or groups of diseases (groups including cancers, cardiovascular diseases, chronic respiratory diseases, upper respiratory infections, and urinary infections). The most common diseases or groups were cancers (n = 2), chronic respiratory diseases (n = 2), diabetes (n = 2), Human Papillomavirus (HPV) (n = 2) and Japanese encephalitis (n = 2). The risk stratification articles mainly focussed on HIV/AIDs (n = 14), with one paper focusing on HIV-associated cryptococcal disease, and others on Ebola, Hepatitis B, malaria, measles, meningitis, and pulmonary tuberculosis. The hotspot analysis articles included eight diseases including pneumococcal disease (n = 2) and one article each on cholera, Japanese encephalitis, malaria, meningococcal meningitis, pulmonary tuberculous and unspecified vaccine-preventable diseases.

**Medicine type**

Overall, articles included the medicines that were drugs (oral regimens; MCDA = 4, risk stratification = 18, hotspot analysis = 3), vaccines (n = MCDA = 3, risk stratification = 4, hotspot analysis = 6) or a combination of drugs and vaccines (MCDA = 1, risk stratification = 0, hotspot analysis = 1) ( Figure 3B). In total, 6 articles (MCDA n = 2, risk stratification n = 3, hotspot analysis n = 1) focused on the introduction of a vaccine (which was new) to their study location. The remaining MCDA vaccine study focused on prioritizing the allocation of a limited vaccine supply within an existing vaccination programme rather than introducing a new vaccine. ^31^ The most common drug regimen was antiretroviral therapy (n = 11). Vaccines included tetanus vaccine, BCG vaccine, group A meningococcal (MenA) conjugate vaccine, and pneumococcal conjugate (PCV) vaccine, and vaccines for Japanese encephalitis (JE), dengue, human papillomavirus (HPV), influenza, typhoid, cholera, and rotavirus.

**Target population**

Target populations varied considerably including the general population, specific age groups (adults, children) and defined patient groups (see Table 2). Selected articles report variable populations depending on the medicine type (i.e. drug or vaccines) or location (e.g. municipalities).

**Table 2.** Summary of medicine target population and type by analytical method.

Target population	MCDA		Risk stratification		Hotspot analysis	
	Drug	Vaccine	Drug	Vaccine	Drug	Vaccine
General Population (All)	5	2	0	3	1	0
Variable by Intervention	0	1	0	0	0	0
Variable by Location	0	0	0	1	0	0
Adult (non-patients)	0	1	3	0	0	1
Children (non-patients)	0	0	1	0	2	4
Patients (All)	0	0	1	0	1	1
Patients (Adult)	0	0	11	0	0	0
Patients (Children)	0	0	5	0	0	1
Couples	0	0	2	0	0	0

Note: Selected articles had more than one target population depending on the method.

For example, hotspot analysis study populations comprised children ( *n* = 6/10; to examine BCG and PCV vaccine coverage, distribution of zero-dose childhood vaccination status, and deworming distribution or uptake), a general population ( *n* = 1; to examine malaria distribution), adults ( *n* = 1; to examine tetanus vaccine coverage), and patient groups ( *n* = 2/10; to examine cholera risk and meningococcal meningitis distribution).

**Implementation stage**

The stage of the medicine translation varied by each analytical method and included:

1. Before the activity begins i.e. pre-implementation to plan, prepare, and/or decide on the choice of medicine, location, or subgroup.
2. During the activity i.e. implementation process to monitor progress, assess and/or adjust.
3. After the activity i.e. post-implementation to evaluate outcome and/or impact.

All eight MCDA articles focused on the before/pre-implementation stage. Risk stratification and hotspot analysis articles reported before, during, and after implementation stages and combinations of these depending on the aims of the study. For hotspot analysis for example, the choice before implementation (n = 2/10), e.g., identifying high-risk areas for cholera to inform Oral Cholera Vaccine (OCV) deployment ^60^; the choice before and the assessment after implementation ( *n* = 6/10), e.g., assessing BCG vaccine coverage and identifying low coverage areas to inform future vaccine interventions ^55^; or the assessment after implementation only ( *n* = 2/10), e.g., to identify the distribution of deworming medications. ^58^ Figure 3C highlights the trends for each method. Data are summarised in the S1 Appendix in the Dryad Data Repository (DOI: 10.5061/dryad.kwh70rzjp).

**Geographic or administrative scale**

The geographic or administrative level of the study methods described in the articles varied, with national, sub-national, healthcare (including clinical) facility, and community levels being the most reported. Figure 3D highlights that MCDA articles conducted studies at subnational (district n = 5) or national (n = 3) level. Risk stratification articles mainly conducted studies at healthcare facility level (n = 16), compared with national (n = 1), sub-national (n = 5) or community (n = 3) levels. Hotspot analysis studies included community (n = 3) level and variable sub-national spatial units which were generally more specifically defined in line with the specific analysis (district n = 1, enumeration area n = 5, health centre catchment area n = 1).

### MCDA approach and findings

**Summary**

The overall aim of seven of the eight MCDA articles was to develop a MCDA framework that could be used to compare and rank multiple interventions – in this study, medicines – to support an informed decision about which option to use. The remaining MCDA article instead aimed to develop a MCDA framework to prioritise individuals (or population groups) for COVID-19 vaccination during vaccine shortages. ^31^ This study more closely resembles risk stratification than intervention prioritisation, as it used MCDA to identify individuals at highest risk for priority vaccination rather than to rank competing vaccines directly. Information summarised in the S2 Appendix in the Dryad Data Repository (DOI: 10.5061/dryad.kwh70rzjp).

Of the articles that ranked interventions, six ranked their medicines or vaccines primarily regarding health impact and one to support procurement. One article focused on developing an MCDA framework for orphan medicinal products based on the EVIDEM framework.

There were three articles that provided visual images or decision-support steps of the process in their study, which they describe as ‘Development and application of a multi-criteria priority setting algorithm in Iran’, ^25^ ‘Formulary listing framework’ ^26^ and “MCDA tool development process”, ^28^ and are available in the S2 Appendix in the Dryad Data Repository (DOI: 10.5061/dryad.kwh70rzjp). Each article followed a series of steps to help ensure each intervention was assessed comprehensively. The steps are briefly described here and outlined as a template in Figure 4, with an example from an article included to demonstrate its application.

1. Define aim of study and use of MCDA
2. Choose intervention or medicines to be evaluated and ranked
3. Select criteria and methods for ranking medicines
4. Score interventions or medicines against each criterion to develop on overall ‘performance score’ or ‘performance matrix’
5. Weight each criterion based on their relative importance or policy priority
6. Rank intervention or medicine by combing performance score/matrix and criteria weighting outcomes

**Figure 4.**
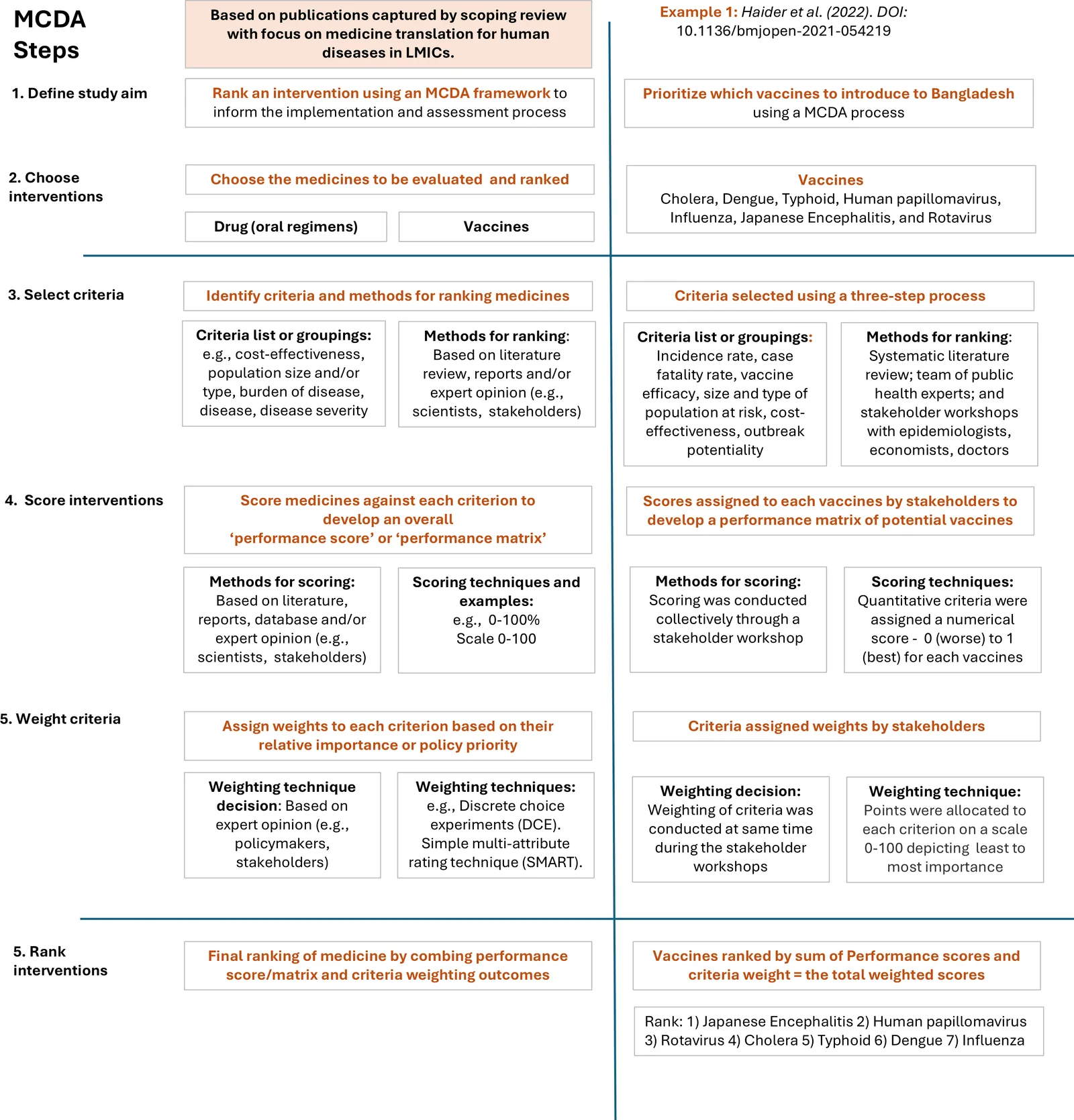
MCDA steps summarised and presented as a preliminary template, with an example.

**Selected criteria**

Each article began commonly by selecting the relevant criteria, which involved expert opinion including consultation of policymakers, stakeholders, and a scientific field expert. Seven articles also conducted a comprehensive review of relevant literature. ^25^^–^^28^^,^^30^^–^^32^ Of the MCDA articles that ranked interventions, the number of criteria ranged from 3 to 19 and criteria were broadly categorized into five groups ( Table 3). The remaining MCDA article that instead ranked individuals for COVID-19 vaccination prioritisation used 10 criteria instead related to demographics and clinical risk factors, including age and obesity. ^31^ The criteria listed for each article and a summary of how they potentially relate to the EVIDEM framework are summarised in the S2 Appendix in the Dryad Data Repository (DOI: 10.5061/dryad.kwh70rzjp).

**Table 3.** Broad criteria groups for intervention ranking.

Grouping of criteria	Criteria listed in articles	Reference
Cost-effectiveness/economic benefit	Cost-effectiveness of the intervention, Cost-effectiveness of the drugs, Cost-effectiveness, macroeconomic benefit	^25^^–^^28^^,^^30^^,^^32^
Size of population (at risk/intervention impact)	Number of people potentially affected by the intervention, Size of population at risk	^25^^,^^27^
Type of population (at risk/intervention impact)	Variations among income levels (prevalence differences between income levels: equity/income-level variation), The social class affected by the disease, Type of population at risk, Target groups of interventions, Gender of target groups	^25^^–^^27^^,^^29^
Burden of disease	Attributable burden, Incidence rate, Case fatality rate, Outbreak potentiality, Premature deaths averted per year, Incident cases prevented per year	^27^^,^^30^^,^^32^
Quality of evidence	Quality of evidence of intervention effectiveness, Quality of evidence	^29^^,^^30^

**Scoring intervention**

Of the seven intervention-scoring articles, six articles scored each intervention across all finalised criteria included in their study. Scoring involved examining relevant data sources about each intervention including literature, reports and databases in all six articles, and consulting expert opinion in four articles. ^26^^–^^28^

For example, in Haider et al., ^27^ data on the potential vaccines was gathered from databases and reports from key public health and research organisations in Bangladesh. A ‘performance matrix’ was constructed from this data, which presents the data for each vaccine against each criterion [example highlighted in Figure 4]. Similarly, Suwantika et al. ^32^ used the Strategic Multi-Attribute Ranking Tool (SMART) Vaccines framework to score candidate vaccines against predefined criteria using country-specific evidence. Likewise, in Elezbawy et al., ^28^ the performance of each assessed medicine was evaluated across all criteria based on relevant literature and expert input, with intervention scores ranging from 0% to 100%. Scoring interventions across each criterion develops a ‘performance matrix’, which features performance scores that capture how well each intervention meets each criterion.

**Weighting criteria**

Weighting the criteria involved expert opinion in all six articles, however the weighting methods differed: two articles used discrete choice experiments, ^26^^,^^29^ one article used pairwise comparison, ^25^ one article used direct weighting methods (point scale), ^27^ one article used stakeholder-derived weighting within the SMART Vaccines framework, ^32^ one article used the SMART (simple multi-attribute rating technique) method combined with swing-weighting technique, ^28^ and finally one article used the five-point weighting and two-step percentile distribution method separately. ^30^ Two articles also included non-expert opinions, for example, people living with HIV/AIDs and village health volunteers and public opinion, respectively, within the weighting process. ^29^^,^^30^

The COVID-19 vaccination study similarly elicited expert-derived weights for demographic and clinical risk factors, with obesity and age receiving the highest weighting. ^31^

**Intervention rank**

Of the seven intervention-ranking articles, six articles determined a final priority rank order for each medicine. Five of the six articles calculated the weighted scores (performance score x criteria weight) for each intervention across all criteria, and the sum of these scores was used to determine the overall priority ranking for interventions. ^25^^–^^28^

For example, in Haider et al. ^27^ using the additive method, the scores of each vaccine corresponding to each criterion (performance scores in the performance matrix) were multiplied by the weight of each criterion to calculate the total (weighted) scores of each potential vaccine. The vaccines were ranked based on the total scores of each vaccine (summed weighted scores across all criteria for each vaccine), as shown by the final intervention ranks in Figure 4. This same process was carried out in Elezbawy et al., ^28^ where for each criterion, the performance score achieved is multiplied by the corresponding weight of the criterion to calculate the final weighted score for each criterion, with these weighted scores then summed to give final weighted scores on which medicines could be ranked. Similarly, Suwantika et al. ^32^ combined criterion scores with stakeholder-derived weights within the SMART Vaccines framework to generate overall scores and rank candidate vaccines.

The remaining intervention-ranking article instead combined a binary performance matrix with regression coefficients (weights) from the discrete choice experiments and transformed these scores into ‘probabilities of selection’ using a logistic regression model, which were used to determine final priority intervention ranks. ^29^

Finally, the MCDA study prioritising individuals for COVID-19 vaccination, rather than ranking interventions directly, used weighted demographic and clinical risk scores to prioritise vaccine allocation. ^31^

### Risk stratification approach and findings

**Summary**

Overall, the aims of the 22 risk stratification articles were highly variable, but broadly delineated risk levels that could inform medicine translation. Of these, 18 articles used individual level stratification to assign risk levels to patients in clinical contexts, while the remaining 4 articles used population level stratification to assign risk levels to areas. Notably, 6 articles directly used the term ‘risk stratification’ to describe their methodology, with common alternative terms, including ‘risk scoring,’ ‘risk assessment,’ ‘risk classification’ or ‘risk prediction.’ The aims of each article and type of risk stratification used are summarised in the S2 Appendix in the Dryad Data Repository (DOI: 10.5061/dryad.kwh70rzjp).

Most articles developed their own risk stratification approach, others adapted or directly used previously developed risk stratification systems without alteration, as summarised here.

- 3 articles stratified groups by a single variable prior to risk analysis (basic risk stratification).
- 5 articles used an already developed risk stratification system/score without alteration.
- 15 articles developed their own risk stratification system/score.
- 2 articles adapted an existing risk stratification system/score.

There were five articles that provided visual images or decision-support steps of the process in their study which they describe as Simplified prediction model formatted as scoring system, ^52^ CD4-independent and CD4-dependent clinical score cards, ^33^ HIV-1 risk score worksheet, ^38^ Summary of quantitative phase of District Prioritization Tool evaluation ^35^ and Example of categorical risk stratification without scoring, ^47^ and are available in the S2 Appendix in the Dryad Data Repository (DOI: 10.5061/dryad.kwh70rzjp).

Each article followed a series of steps to assess the medicine which are briefly described here and outlined as a template in Figure 5 with an example from an article included to demonstrate its application.

1. Define aim of study and use of risk stratification
2. Define outcome measures by defining level, groups, or categories to delineate risk
3. Determine risk factors by identifying and selecting multiple variables that potentially influence the outcome
4. Determine risk score of each factor using a scoring system or probabilities [model]
5. Stratify risk and identify target groups or categories based on score outcomes and cut-off thresholds

**Figure 5.**
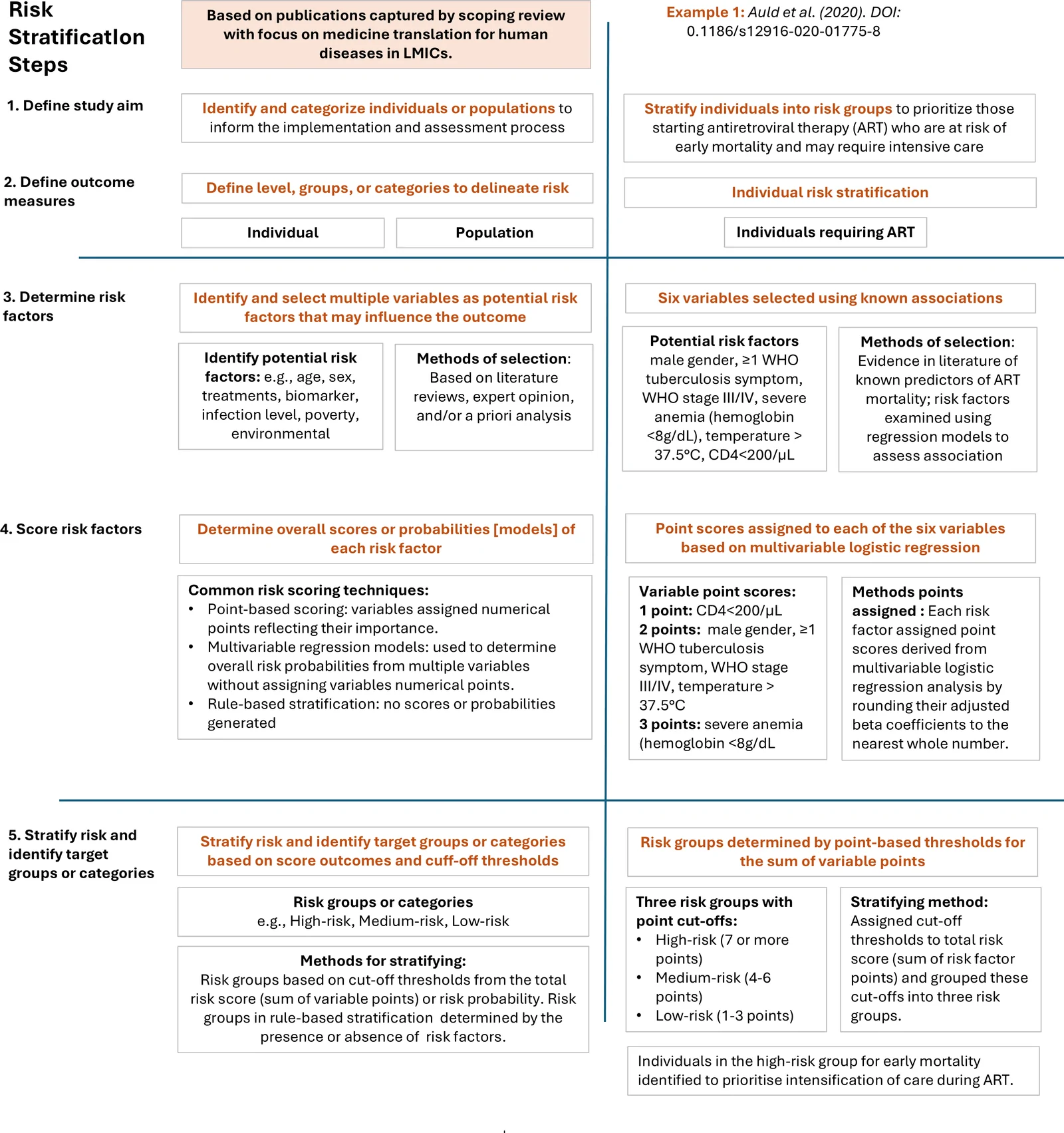
Risk stratification steps summarised and presented as a preliminary template, with an example.

**Define variables and data sources**

In total, 18 articles used a finalised list of variables as potential risk factors that captured different dimensions of risk. The number of factors used ranged from 2 to 19, including a set of selected sub-risk factors. A summary of the number and list of factors used for each risk stratification is shown in Table 4, which were commonly chosen from known literature of predictors. Data were obtained from a variety of sources including clinical field studies, international epidemiological databases, national surveillance data, institutional medical records. and un/published literature. The full list of factors and data sources are summarised in the S2 Appendix in the Dryad Data Repository (DOI: 10.5061/dryad.kwh70rzjp).

**Table 4.** Summary of common variables (potential risk factors) used for risk stratification.

Individual/clinical risk stratification	Reference
Age	^38^^,^^40^^–^^44^^,^^46^^,^^49^^,^^50^^,^^52^
Sex	^33^^,^^37^^,^^42^^,^^49^^,^^50^
Time on treatment	^39^^,^^44^
Adherence to treatment	^40^^,^^41^^,^^48^
Biomarkers	^33^^,^^36^^,^^37^^,^^39^^–^^42^^,^^44^^,^^45^^,^^47^^,^^50^
Viral/Bacterial load	^38^^,^^41^^,^^42^^,^^47^^,^^48^^,^^51^
Social/Environment factors	^38^^,^^41^^,^^43^^,^^49^^,^^52^

Population risk stratification	Reference
Epidemiological risk	^34^^,^^35^
Population density	^35^^,^^53^
Programme capacity/ability	^34^^,^^35^

Note: Biomarkers included CD4 cell count, haemoglobin level, protein level, blood neutrophil level and imaging (chest radiograph).

**Score system**

In total 19 articles used scoring approaches that we broadly group into four categories, shown in Table 5 with diseases and countries of focus. Each approach is summarised here with selected examples.

- Point- based risk score – 11 articles assigned an overall risk score (to individuals or areas) by combining data across variables. Each variable was assigned points (integer value) relative to its importance i.e. variables with stronger impacts given more points, with variable impacts sometimes determined through multivariable regression analysis. Binary variables (yes/no) were assigned higher risk a positive point (i.e.1), while the lower risk typically scored a 0. Continuous variables were commonly categorized by cut-off thresholds. Point-based example include Auld et al., ^33^ where a combination of clinical thresholds - such HIV disease stage (III/IV = 2 points vs I/II – 0 points)) and haemoglobin (none = 0 points, mild/moderate = 1-2 points, and severe = 3 points) - were categorised, and then a patient’s overall score calculated by totally all points across all variable, and grouping into low-risk (<4points), moderate-risk (4-6 points) and high-risk (≥7 points) categories (in Figure 5).
- Probability-based risk models – did not develop risk scores but instead incorporated each variable into models that determined overall risk probabilities for an individual or area. For example, in Wells et al. ^53^ human mobility, poverty and population density were used to estimate overall probabilities of an Ebola infection across 219,152 spatial units.
- Machine-learning risk scores – calculated overall risk scores through ensemble supervised machine learning. For example, in Koss et al. ^49^ an ensemble machine learning approach was used to derive a risk score based on HIV seroconversion and socio-demographic data from the first two years of the SEARCH test-and-treat trial.
- No risk scores or risk probabilities calculated – this group used categorical variables and did not develop risk scores or probabilities from their stratification frameworks.

**Table 5.** Summary of the scoring approach to defining risk by region and disease focus.

	Point-based risk scores	Probability-based risk models	Machine-learning risk scores	No risk scores or risk probabilities calculated
Number of Articles	11	3	1	4
WHO Region	AFR, AMR, SEAR	AFR	AFR	AFR, AMR, SEAR, WPR
Diseases of focus	HIV/AIDs, Meningococcal meningitis (serogroup A), Non-typhoidal Salmonella (NTS)	HIV/AIDs, HIV-associated cryptococcal meningitis, Ebola	HIV/AIDs	HIV/AIDs, measles, malaria, pulmonary tuberculosis
List of Articles	^33^^,^^35^^,^^37^^,^^38^^,^^40^^–^^44^^,^^50^^,^^52^	^39^^,^^45^^,^^53^	^49^	^34^^,^^47^^,^^48^^,^^54^

Note: The three articles defined as basic stratification are not included.

**Categorise**

Overall risk groups or categories were based on a broad risk stratification framework. Specifically, the articles that used a point-based scoring system, used cut-off value thresholds for the total accumulated risk score. The articles that used machine-learning based risk scores or probability-based risk models, also used these outputs to define risk groups or categories. Wells et al. ^53^ used risk probability cut-off values to define categories: High-risk (0.05 or more) Moderate-risk (between 0.01 and 0.05), Low-risk (between 0 and 0.01), and Samuels et al. ^13^ produced three risk categories that were defined by tertiles of predicted risk: High-risk, Medium-risk, Low-risk. Risk groups are summarised in the S2 Appendix in the Dryad Data Repository (DOI: 10.5061/dryad.kwh70rzjp).

The four articles that did not assign variable risk scores or risk probabilities defined risk groups or categories using different measures such as a rule-based stratification or the presence or absence of a specific criteria. For example, Chang et al. ^47^ used clinical categorical variables (i.e., Xpert MTB/RIF Ct < 18 and Chest radiograph disease extent ≥ 50) to triage tuberculous patients and stratify disease phenotypes into easier, moderately-hard and harder-to-treat groups. Bose et al. ^34^ used a ‘logical decision-making framework’ to categories areas as ‘Go’ or ‘No-Go’ for outbreak response immunisation without calculating risk scores or probabilities for these areas. Risk groups are summarised in the S2 Appendix in the Dryad Data Repository (DOI: 10.5061/dryad.kwh70rzjp).

### Hotspot approach and findings

**Summary**

Overall, the aims of the six hotspot analysis articles were to investigate the spatial and temporal dynamics of infectious disease distribution or risk ( *n* = 3/10), drug distribution ( *n* = 2/10), or vaccine coverage ( *n* = 5/10). Outcome measures comprised: distribution or risk of meningococcal meningitis, *Plasmodium falciparum* malaria, and cholera; coverage or uptake of BCG, tetanus, pneumococcal or childhood vaccinations; and distribution or uptake of deworming. Data are summarised in the S2 Appendix in the Dryad Data Repository (DOI: 10.5061/dryad.kwh70rzjp).

Broadly, each article followed a series of steps, briefly described here and outlined as a template in Figure 6, with two examples included.

1. Define the aim and use of hotspot analysis.
2. Define the outcome measure.
3. Define variables of interest, specifically the study area, target population, medicine/ intervention or disease, and stage of medicine/intervention implementation.
4. Determine the geospatial unit and data type, categorised broadly into point (e.g., latitude and longitude coordinates) or polygon data (e.g., administrative division boundaries).
5. Identify data sources, including open-access research data or government, multi-lateral agencies, and non-governmental data resources.
6. Select the hotspot analysis methods and Geographic Information System (GIS) software.
7. Generate and map output measures and/or thresholds.
8. Interpret outputs to inform targeting of medicines/interventions.

**Figure 6.**
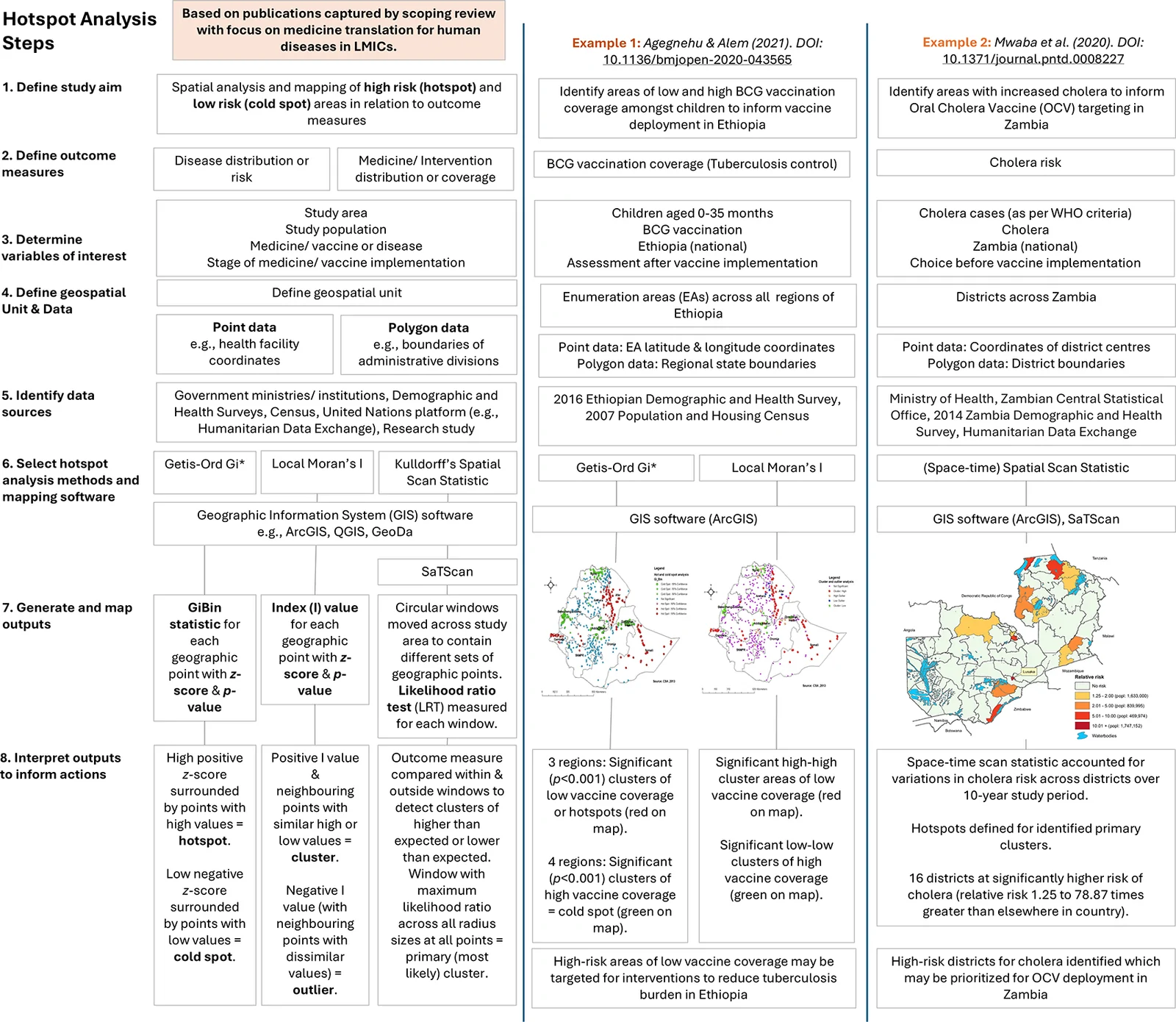
Summary and preliminary outline of the steps involved in a hotspot analysis process, with two examples.

**Variables**

A wide range of independent variables or potential risk factors were examined, and frequently included different levels or categories mainly at an individual (e.g. age, education, vaccination status, clinical episode, clinic visits, drug uptake), household (e.g. wealth, media use), and/or community level (literacy, connectivity to health facility). In addition, variables related to the geographic characteristics (e.g. urban/rural, poverty level), vectors (e.g. human biting rate), and temporality (e.g. month) were examined. Data are summarised in the S2 Appendix in the Dryad Data Repository (DOI: 10.5061/dryad.kwh70rzjp).

**Methods and software**

Overall articles specified using Getis-Ord Gi* ( *n* = 6/10), Kulldorff’s spatial scan statistic ( *n* = 2/10), and Local Moran’s I ( *n* = 1) statistical methods or a combination of these to conduct the analysis which are highlighted in the S2 Appendix (Dryad Data Repository DOI: 10.5061/dryad.kwh70rzjp). One article did not specify ‘hotspot analysis’ but applied spatial scan statistic to identify high-risk spatial clusters of cases. ^58^

Geographical information systems (GIS) software including ArcGIS and SaTScan software were used to map hotspot and cold spot areas, and to conduct spatial scan statistic, respectively. Spatial analytical techniques used in conjunction with specified hotspot analysis methods comprised techniques for spatial autocorrelation (Global Moran’s I), cluster and outlier analysis (Local Moran’s I), spatial scan statistical analysis (Kulldorff’s spatial scan statistic), and spatial interpolation methods (Kriging, inverse distance weighting). S2 Appendix presents mapping output examples from selected articles and available in the (Dryad Data Repository DOI: 10.5061/dryad.kwh70rzjp).

**Risk classification**

The Getis-Ord Gi* method used z-scores and p-values, which were also presented in selected articles as Gi_Bin classes (z-scores at the 90%, 95%, and 99% confident intervals), to identify areas of significant spatial clustering. For example, Agegnehu and Alem ^55^ exploring spatial variation in BCG vaccination in Ethiopia found significant clusters (hotspots) of low coverage in three regions (Gi-Bin = 3; z-scores 3.95-4.12, and <0.001 p value) (see Figure 6).

The spatial scan statistic method used Log Likelihood Ratio (LLR) to detect significant clusters (hotspots), followed by a calculation of relative risk to determine the magnitude of risk. For example, Mwabe et al. ^60^ identified district-level cholera hotspots in Zambia, with relative risks ranging from 1.25 to 78.8 in high-risk areas see ( Figure 6).

The Local Moran’s I method used z-scores and p-values to identify: i) a cluster (hotspot) - high positive values, ii) an outlier - negative values, or iii) no spatial association - values near zero. For example, Belay et al. ^57^ found hotspots of low tetanus vaccination coverage in Ethiopia, displayed in a map of dots representing 90%, 95%, and 99% confident intervals, which correlate with Z-score thresholds 1.65, 1.96 and 2.58 respectively. The map is shown in the collation of hotspot examples in the S2 Appendix (Dryad Data Repository DOI: 10.5061/dryad.kwh70rzjp).

### Grey literature

**Conference abstracts**

Annual ASTMH conference abstract books from 2015 to 2024 were reviewed for abstracts describing the three analytical methods. From an initial pool of 39 abstracts flagged by the keyword search, 12 met the inclusion criteria for interventions. Narrowing further to medicine-specific interventions, only 6 abstracts remained for final analysis, from which only three ASTMH abstracts were identified and included as they described the use of risk stratification ( *n* = 1) and hotspot analysis ( *n* = 1) methods (see S3 Appendix for details of abstracts available in the Dryad Data Repository DOI: 10.5061/dryad.kwh70rzjp). Both abstracts explicitly stated terms relevant to the keyword search (‘risk prediction’ and ‘spatial analysis’ respectively) and described analytical methods consistent with our standard definitions of risk stratification or hotspot analysis with a focus on malaria, schistosomiasis and soil-transmitted helminths. No abstracts describing MCDA with a focus on medicine translation for human diseases were identified. Relevant characteristics of the 39 abstracts are summarised in the S3 Appendix (Dryad Data Repository DOI: 10.5061/dryad.kwh70rzjp). Due to the limitations of abstract data presented and lack of detailed description of the analytical methods used, identified abstracts have not been included in the PRISMA flow diagram but the stepwise process is shown in S3 Appendix (Dryad Data Repository DOI: 10.5061/dryad.kwh70rzjp).

**National and regional institutions**

The following global and regional institutional webpages and online research libraries were reviewed: WHO webpage; WHO region webpages for WHO AFRO, AMRO, SEARO and WPRO (n = 4); Africa CDC; and the African Journals Online. National institutional webpages (n = 16), scientific journal webpages (n = 12), and online research libraries (n = 3) were reviewed across eight countries within WHO AFRO (Ethiopia, DRC, and Nigeria), and WHO AMRO (Brazil, Colombia, Mexico, Argentina, and Peru). Data are summarised in the S3 Appendix in the Dryad Data Repository (DOI: 10.5061/dryad.kwh70rzjp).

No national or regional level resources describing the three analytical methods in the context of medicine translation for human diseases in LMICs were identified. A common reason for resource exclusion was that medicine translation was not described clearly in relation to the human diseases of focus. Major search challenges comprised: language barriers (e.g., potential resources from WHO AMR and AFR countries were only available in Spanish, Portuguese and/or French); access restrictions to research archives; and limited description of methodology in abstracts.

## Discussion

This review highlights that the three data analytical methodologies have been used across five global regions, in 36 countries for the purpose of optimising medicine translation. Overall, there was an increasing trend in the use of these methodologies, especially in Africa. This suggests that there is interest, knowledge, and capacity that could be developed further with specific data science training including skills in using visualization to improve public health decision-making. ^65^^–^^67^ In addition, increasing locally led research and publications from LMIC researchers may help address imbalances in authorship and create greater opportunities for engagement with national programmes. ^68^ Strengthening local research leadership may also enhance the relevance and uptake of evidence. However, achieving effective and sustainable evidence translation also requires deliberate efforts to align research activities with policy priorities and local health needs. ^69^^–^^71^

A diverse range of diseases were studied; however, most were communicable infectious diseases, specifically HIV/AIDs. This may be due to the high level of funding for the ‘Big Three’ diseases - HIV/AIDS, tuberculosis, and malaria, and the related international networks and expertise. ^72^ This group of diseases aligns with SDG 3 targets, and focussed analytical methodologies that help direct interventions to advance global health targets. ^6^ Most articles focused on the drug and vaccine pre-implementation stage to delineate risk and/or decide on the appropriate intervention, however, applying these methodologies more broadly to the during and after implementation stages can demonstrate outcomes and value for money to donors. ^73^

Most articles used different approaches even when applying the same analytical method, which limited comparability and synthesis of the findings. The variability may stem from a lack of clear guidelines or examples to guide their work, which is a recognised challenge for evidence-based practices. ^74^^,^^75^ Our findings are consistent with broader LMIC priority-setting literature. Kaur et al. ^76^ reported substantial heterogeneity in the criteria, methods and frameworks used for resource-allocation decisions, despite common consideration of health benefits, equity and cost-effectiveness. Similarly, we found considerable variation in the application of MCDA, risk stratification and hotspot analyses, highlighting a continued need for greater methodological standardisation. Our literature search found few examples of templates, visual images or decision-support tools that could be generalised or were intended for wider use. However, we were able to identify broad steps of each methodology, which are set out in Figures 4- 6. These may be used as a basis to develop practical standardised approaches to help ensure consistency, and comparability across various aspects, such as regions, diseases, interventions and populations. The templates presented in Figures 4- 6 are intended as broad starting points to support the understanding and planning of these prioritisation techniques, rather than as comprehensive, definitive, or validated workflows. Given the methodological heterogeneity identified across included studies, they should be interpreted as illustrative frameworks that can be adapted to specific contexts and decision-making needs. These templates are therefore intended as practical resources to be used alongside established methodological guidance, such as existing MCDA guidance, which highlights that methodological choices should be guided by the objectives of the analysis, the decision context, and stakeholder preferences. ^77, 78^

The findings also have practical implications for policy and programme implementation. The broad templates developed in this review provide an initial roadmap for selecting and applying prioritisation approaches but should be further refined into practical decision-support resources. Future work could translate these templates into user-friendly guidance, checklists, or decision-support tools that assist Ministries of Health, National Immunization Technical Advisory Groups (NITAGs), Health Technology Assessment (HTA) agencies, and development partners in selecting and implementing appropriate prioritisation methods according to the decision context, available data, technical capacity, and programme objectives. This recommendation is consistent with findings from a recent scoping review by Atwal et al. ^79^

MCDA articles broadly had the most standardised methodology, which may be because it had established frameworks to refer to, such as EVIDEM, to guide implementation. ^13^^,^^14^ Despite this, we found much variation in the type and number of criteria chosen, and the techniques used for scoring and weighting. This variation may reflect differences in country-specific priorities and values, particularly regarding non-clinical and economic considerations. Risk stratification was the most broadly applied across all three implementation stages, with a diverse geographical scope and terminology. It was also found to not be specifically referred to a risk stratification and the processes had a general lack of standardisation. Hotspot analysis followed standard spatial analytical methods, however, how these were used varied considerably. Grey literature yielded little evidence suggesting that these methodologies are not routinely or openly used in policy and practice for medicines. ^2^^,^^3^ However, our grey literature search results did identify topic areas where these methods are being applied, highlighting that there is potential for further development in medicine translation if necessary.

The main limitations to this review include the methodological variability which limited the synthesis of findings; lack of established templates, visual aids, or decision-support tools to use as a basis to develop a more structured process; no comparison with higher income countries where practical approaches may exist and provide insights and comparisons; and non-exhaustive grey literature searches, which were limited to resources in English. Notwithstanding these limitations, this review provides key insights into the use of these three data analytical methodologies in LMICs for medicine translation. Knowledge gaps were identified, the need for capacity development was highlighted, and outcomes may further inform the development of templates as decision-support tools.

Another limitation is that our grey literature search did not include Health Technology Assessment (HTA) agency websites. Given that MCDA and priority-setting are increasingly used within HTA processes, agencies such as the Health Intervention and Technology Assessment Program (HITAP, https://www.hitap.net/en/home/), the International Decision Support Initiative (iDSI, https://www.idsihealth.org/), and national HTA bodies in Africa and Latin America may host relevant reports, technical guidance, or case studies that are not indexed in bibliographic databases. Future reviews should consider systematically searching these sources to improve the comprehensiveness of evidence retrieval.

## Conclusion

The MCDA, risk stratification, and hotspot analysis methodologies provide a structured basis for further developing practical templates and decision-support tools to enhance the evidence translation for medicines. However, there is a need to raise awareness, strengthen capacity, and advocate for their use in LMICs to facilitate a more standardised and transparent process and help make progress towards the SDG 3 Good Health and Well-being and the specific related global targets.

## Data Availability

No data are associated with this article. All data produced in the present work are contained in the manuscript or supplementary material. All supplementary material can be found on the Dryad Digital Repository in a file entitled “Supplementary materials for Characterizing multi-criteria decision analysis, risk stratification, and hotspot analysis methods to optimise disease policymaking and evidence translation for medicines in low- and middle-income countries: A scoping review.” (DOI: 10.5061/dryad.kwh70rzjp). Files include: S1 PRISMA-Checklist, S1 Appendix, S2 Appendix, S3 Appendix.
